# Achieving waste to energy through sewage sludge gasification using hot slags: syngas production

**DOI:** 10.1038/srep11436

**Published:** 2015-06-15

**Authors:** Yongqi Sun, Jinichiro Nakano, Lili Liu, Xidong Wang, Zuotai Zhang

**Affiliations:** 1 Department of Energy and Resources Engineering, College of Engineering, Peking University, Beijing 100871, P.R. China; 2 URS Corp., PO BOX 1959, Albany, OR 97321, USA; 3 Beijing Key Laboratory for Solid Waste Utilization and Management, College of Engineering, Peking University, Beijing 100871, P.R. China

## Abstract

To relieve the environmental issues of sewage sludge (SS) disposal and greenhouse gas (GHG) emission in China, we proposed an integrated method for the first time to simultaneously deal with these two problems. The hot slags below 920 °C could act as a good heat carrier for sludge gasification and the increasing CO_2_ concentration in CO_2_/O_2_ atmospheres enhanced the production of CO and H_2_ at 400–800 °C. Three stages of syngas release were clearly identified by Gaussian fittings, i.e., volatile release, char transformation and fixed carbon reaction. Additionally, the effect of sulfur retention of slags and the synergy effect of the stabilization of toxic elements in the solid residuals were discovered in this study. Furthermore, a novel prototype of multiple industrial and urban systems was put forward, in which the produced CO + H_2_ could be utilized for direct reduced iron (DRI) production and the solid residuals of sludge ash and glassy slags would be applied as cementitious materials. For a steel plant with an annual production of crude steel of 10 million tons in China, the total annual energy saving and GHG emission reduction achieved are 3.31*10^5^ tons of standard coal and 1.74*10^6^ tons of CO_2_, respectively.

In order to meet the urgent environmental challenges, the integrated recovery and reasonable disposal of the wastes produced by the industrial and urban sectors have increasingly become important. Amongst them, hot slags from the steel industry^1^ and sewage sludge (SS) from the wastewater treatment plants^2^ are two kinds of particular waste residuals required to be timely and efficiently disposed. The heat recovery from hot slags, at ~1550 °C before tapping, is believed to represent the last potential of energy efficiency improvement in the steel industry^3^,^4^. In China, the steel industry produced more than 710 million tons of crude steel in 2012 alone^5^ and accordingly 210 million tons of blast furnace slags (BFS), the total waste heat of which was equivalent to 16 million tons of standard coal. However, the recovery ratio of waste heat from slags was less than 2% in 2006^6^; hence the energy recovery from slags is a major problem to be addressed.

Conventionally, the water quenched slags, with amorphous structure and high hydraulicity, can be used as cementitious materials^4^,^7^, which can partially substitute the CaO consumption, decrease the need in the calcination of CaCO_3_, and therefore reduce the CO_2_ emission in the cement industry. However, a series of problems including water consumption, H_2_S and SO_2_ release and energy waste, are caused through the waster-quenching method^3^,^4^. Thus, to exploit integrated routes to recover both the waste heat and the material resources from slags is of great significance, from point of which a method of multiple stages is scientific. The liquid slags should be first rapidly quenched to avoid crystallization and form glassy state, and the waste heat of the solid slags can be then recovered by various strategies, such as chemical methods, which is actually the idea of our previous studies^8^,^9^.

The discontinuous waste heat of slags can be transferred into the continuous chemical energy in syngas through chemical methods^10^, amongst which methane reforming^11^,^12^ and coal gasification^13^,^14^ have been extensively investigated. It has been found that the hot slags even exhibit some catalytic effect on the coal gasification, for the reason that the coal gasification is generally performed at 1300–1500 °C^13^ and 950–1150 °C^14^ where crystal can form in the slags. However, in views of slag material recycling, the crystallized slags should be avoided for the subsequent application as cementitious material, which is, in fact, one of the main objectives of this study. To achieve the heat recovery and material recycling of slags, an emerging and integrated method, namely sludge gasification, was explored in this study. The accelerating urbanization in China causes a continuously increasing generation of SS, the effective utilization and disposal of which are becoming a significant environmental task^2^,^15^. In 2012, ~63.6 billion m^3^ sewage was discharged in China and to treat the sewage, more than 6.55 million tons of dry SS was generated^16^. Many routes are adopted to dispose the SS, including incineration and gasification^17^,^18^. Through high temperature treatment, the toxic organic compounds and pathogens are destroyed and the energy in sludge is partially recovered. Furthermore, the heavy metals (HMs) in the ash, such as Pb, Cu, Cr, Ni, Cd and Hg, are quite well-immobilized and resistant of leaching^18^,^19^; thus, the ash can be applied in the Portland cement manufacturing to substitute CaO^18^,^20^. There is a great advantage that the mixture of the slags and sludge ash after sludge gasification is utilized in the cement industry and the waste residuals are effectively treated, which was actually one of the reasons why this study was motivated.

Currently, the sludge gasification can be performed in air or CO_2_/O_2_ atmospheres, with the purpose of producing syngas (CO, H_2_ and CH_4_) or biofuel^21^,^22^,^23^,^24^,^25^. Gasification using air is easy to manage with low cost, while to the point of carbon capture and storage (CCS), gasification using CO_2_/O_2_ is of greater significance^21^,^22^. Moreover, it has been reported that the emission of NO_x_ can be remarkably reduced using CO_2_/O_2_ atmosphere^21^,^23^ and therefore, the atmospheres of air and CO_2_/O_2_ were used in this study to simulate the sludge gasification. Through a series of isothermal experiments, we explored the feasibility of a more integrated system involving heat recovery, materials recycling, and multiple sectors, which may open a pathway for approaching the environmental challenges in China.

## Results

### Cooling path of the BFS

**Figure 1:**
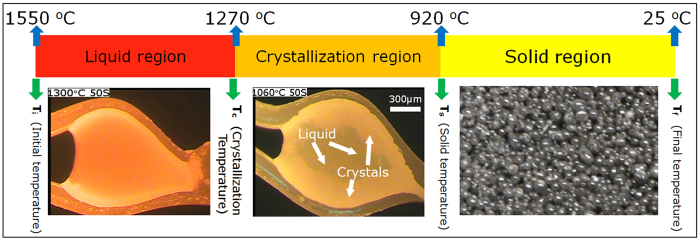
Cooling path of the industrial BFS.

To develop a reasonable waste heat recovery method, an understanding of the cooling path of the slags measured by Single Hot Thermocouple Technique (SHTT) method was fundamentally studied. The initial temperature of the slags was set to be 1550^o^C (denoted as T_i_). As the liquid slag was rapidly quenched to a holding temperature lower than the crystallization temperature, crystal formed in the slag melts, as shown in Fig.1 as an example. The crystallization temperature defined here is the highest temperature at which the crystal forms in the slag melts (denoted as T_b_); 1270 °C in this study. The solidification temperature defined here is the lowest temperature at which crystallization phenomena disappeared (denoted as T_s_); 920 ^°^C in this work. The final temperature of the slags (denoted as T_f_) was assumed to be room temperature (25 °C).

According to these temperature points and the multi-stage control method^8^, the slag cooling path could be divided into three regions, namely a liquid region, a crystallization region and a solid region. In the liquid region, the slags were in the liquid state and should be granulated into small particles. In the crystallization region, the slags should be rapidly quenched to avoid crystalline formation and the waste heat in this region can be extracted by heat exchange between hot slags and working medium, such as air or steam. In the solid region, no crystal was precipitated because of the high resistance of mass transfer and to confirm the glassy phase of slags was significant for the further slag utilization as raw materials in the cement industry. Based on the foregoing cooling path of slags, the sludge gasification experiments in this study were performed in the solid region of slags, i.e., lower than 920 ^°^C, during which slag crystallization could be considerably avoided.

### Calculation of equilibrium syngas production by FactSage

The equilibrium syngas production was calculated by the FactSage software (FactSage 6.3)^26^, in views of theoretical prediction of syngas production and optimization of gasification condition. To simplify the calculation process, the amount of C, H and O atoms were assumed to be 1 mol, 1.82 mol and 0.77 mol, respectively and the reactive CO_2_ and O_2_ varied from 0 mol to 0.5 mol. Assuming that all the reactants including gases were at the same temperature, the higher heating value (HHV) of the syngas was calculated according to the following equation^27^,^28^:


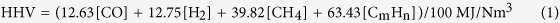


where [H_2_], [CO], [CH_4_] and [C_m_H_n_] represent the molar fractions of H_2_, CO, CH_4_ and hydrocarbon gases with more than one carbon atom in the produced syngas.

**Figure 2:**
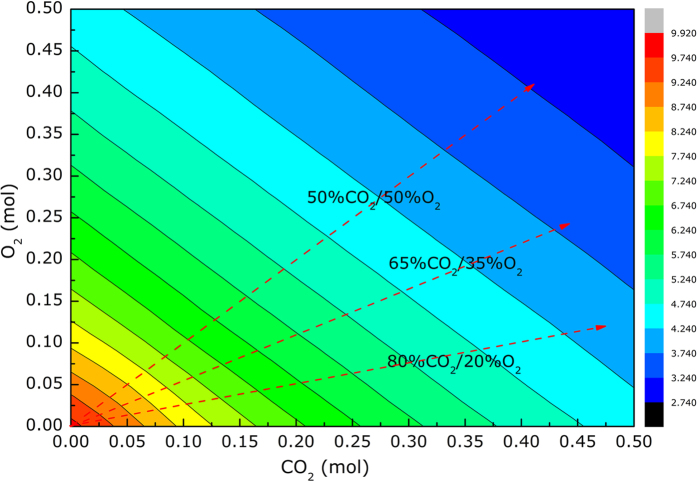
Contour of the syngas HHV (MJ/Nm^3^) in atmospheres of CO_2_/O_2._

As an example, Fig. 2 displays the contour of HHV of syngas produced at 600^o^C and the dash line shows the results under the present experimental conditions, based on which the characteristics related to syngas production could be identified. However, it should be pointed out that, in this study, the content of C_m_H_n_ was so low that it could be ignored for the HHV calculation of the syngas. First, the HHV of syngas decreased with increasing reactive CO_2_ and O_2_ and therefore the key factor to increase the HHV of syngas was to reasonably reduce the reactive agent. Second, an interesting phenomenon was observed that the HHV of syngas kept constant if the total amount of reactive agents was constant, i.e., there was no relation between the HHV of syngas and the ratio of CO_2_/O_2_ as long as the sum of CO_2_ and O_2_ remained constant. For the same amount of CO_2_, on the other hand, the CO_2_/O_2_ ratio influenced the syngas HHV; larger HHV would be obtained by reducing the O_2_ content. During the calculation process, the break of the chemical bonds in the sludge was ignored, which would affect this phenomenon. The results by FactSage pointed out the direction to improve the HHV of syngas, which was of great significance from the point of view of controlling and design of gasification conditions.

### Transient behaviors of syngas release

**Figure 3:**
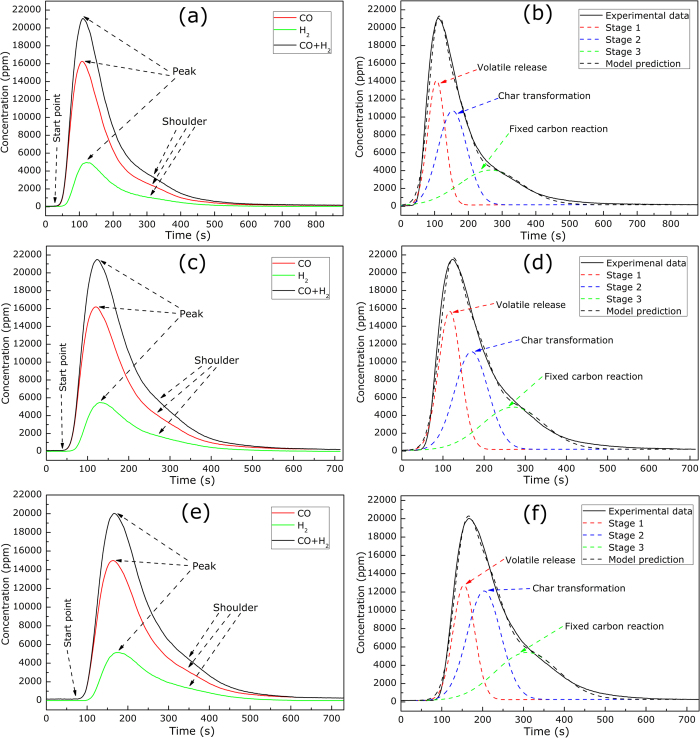
Stages of syngas release based on Gaussian distribution during gasification at 500 ^°^C in 80%CO_2_/20%O_2_ atmosphere for samples (**a**) Syngas production of sample **SS1**, (**b**) Gaussian fitting of sample **SS1**, (**c**) Syngas production of sample **SS2**, (**d**) Gaussian fitting of sample **SS2**, (**e**) Syngas production of sample **SS3** and (**f**) Gaussian fitting of sample **SS3**.

In the present experiments, the evolution of syngas production versus time was detected by gas analyzer and the transient behavior of sludge gasification and gas release was therefore clarified. Fig. 3 shows the transient release of syngas against time at 500 °C in 80%CO_2_/20%O_2_ atmosphere for various samples. In this study, 0.10 g sludge was used with the mass ratios of sludge/slags of 1:0, 1:1, and 1:3, denoted as **SS1, SS2 and SS3**, respectively. Before analysis, three kinds of phenomena observed should be clarified. First, the release of CH_4_ could be detected only in 80%CO_2_/20%O_2_ atmosphere at 600, 700 and 800 °C because the high content of O_2_ improved the oxidization and therefore resisted the release of CH_4_. Thus, the analysis of syngas release was focused on CO and H_2_. Second, the curve shape of transient syngas release for various samples versus temperature did not show significant change as a whole, and therefore the transient curves for gasification at 500^o^C in 80%CO_2_/20%O_2_ atmosphere are presented in Fig. 3 as an example. Third, it should be pointed out that the generation of tar was also observed, the yield of which decreased with increasing oxygen content in the gasifying agent. However, the tar yield was relatively low under the present experimental conditions and it was therefore not discussed in detail.

As can be preliminarily observed, these syngas release curves were mainly composed of one peak, which could be related to the volatile release, and a shoulder in the syngas curves, which could originate from the char reaction. Generally, the sludge gasification and combustion process can be mainly divided into two stages, namely volatile release and char reaction; the former stage was composed of the decomposition of organics and the formation of char, and the latter stage was composed of the transformation of char into fixed carbon and the reaction between fixed carbon and reactive agent^21^,^25^. Because of the relatively low rate of char reaction and the rapid volatile release, the transient curves of syngas release could be divided into a sharp peak on the former side and a flat shoulder on the latter side, and correspondingly the process of syngas release could be at least divided into two stages, i.e., volatile release and char reaction.

In order to further clarify the transient behaviors of syngas release, the transient curves of syngas release were tentatively fitted by Gaussian distributions. This method was first recommended to investigate the biomass gasification process^29^,^30^, which was employed in this study to explore the sludge gasification process. Fig.3 presents the transient curves of syngas release at 500 °C in 80%CO_2_/20%O_2_ atmosphere and the corresponding fitting results, based on which several characteristics could be identified. First, the start point where syngas release was detected was slightly delayed from 40 s for sample SS1 to 45 s for SS2 whereas that of sample SS3 showed a more noticeable delay (75 s). It was assumed that the mass transfer of reactive agent was hindered by the slags. Second, three Gaussian functions could be deduced to fit the transient curves of syngas release, indicating that there were three reaction stages for sludge gasification. Stage 1 was the pyrolysis process, during which the organics was decomposed and released and the char was produced^25^, as illustrated in Eq. (2). There was some tar generated during this stage while the content was quite low. It was reported that the char contained high content of H and O^29^. In stage 2, the char was transformed into fixed carbon, as presented in Eq. (3), during which the syngas was further released. In stage 3, fixed carbon reacted with CO_2_ and more syngas was produced, as described in Eq. (4). Chen *et al*.^29^ also reported the two stage reactions for biomass gasification; compared with the biomass gasification, however, the volatile release stage was weaker and the char reaction stage was stronger in this study because of the lower content of volatile in sludge than that in biomass. In addition, the model prediction composed of three stages (as below) agreed well with the experimental data, which also proved the reasonability of the foregoing analysis.

Stage 1:


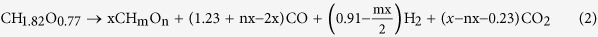


Stage 2:


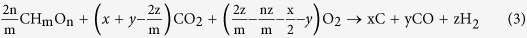


Stage 3:


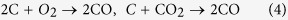


### Syngas production with varying conditions

**Figure 4:**
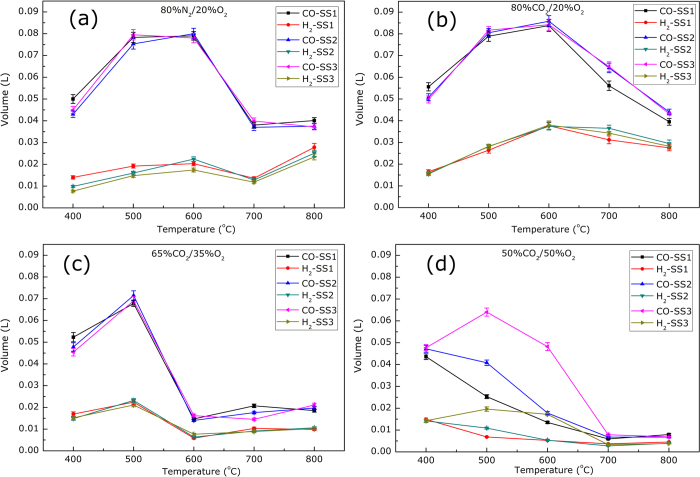
Major Gas yield in terms of temperature per gram of sludge for samples: (**a**) 80%N_2_/20%O_2_, (**b**) 80%CO_2_/20%O_2_, (**c**) 65CO_2_/35%O_2_ and (**d**) 50%CO_2_/50%O_2_.

The total syngas yield per gram of sludge was calculated by the following steps. First, the concentrations of the various gases (CO, H_2_ and CH_4_) in the obtained syngas were measured by the gas analyzer and the transient curves of gas concentrations were therefore obtained. Second, it was assumed that the gas volume kept constant, because the obtained syngas were not remarkably changed in comparison with the total gas volume in this study. According to the total gas volume and the transient curves, the total syngas yield was derived, as summarized in Fig. 4. It should be pointed out that all the gasification experiments were performed at least three times and the average values were used. Additionally, the relative error of value of CO_2_ production was calculated less than 4% and that of H_2_ production was less than 6%. According to Fig. 4, several characteristics could be identified. First, in 80%N_2_/20%O_2_ and 80%CO_2_/20%O_2_ atmospheres, the total syngas production first visibly increased (400–600 °C) and then decreased (600–700 °C) with increasing temperature. From 400 °C to 600 °C, the breakdown of the organics in the sludge was improved by the higher temperature and therefore the syngas yield was increased, while from 600 °C to 700 °C, the oxidization of the sludge, especially the char combustion were dominantly enhanced compared with the lower temperature and consequently more CO_2_ was released; therefore the release of CO and H_2_ were reduced, which was in agreement with previous studies^21^,^31^. It should be noted that in 80%N_2_/20%O_2_ atmosphere, there was an increase of syngas production in the temperature range of 700–800 °C, which could result from the homogenous reactions of CO_2_/CH_4_. In views of the syngas yield, the optimum temperature of sludge gasification was 600 °C in 80%N_2_/20%O_2_ and 80%CO_2_/20%O_2_ atmospheres. Second, in 65%CO_2_/35%O_2_ and 50%CO_2_/50%O_2_ atmospheres, the variation trend of syngas production was different. The syngas yield considerably increased below 600 °C and then decreased with increasing temperature. Because of the high content of oxygen, the sludge oxidization, especially the char combustion was pronouncedly enhanced with increasing temperature and therefore more CO_2_ was formed, finally resulting in the reduction of CO and H_2_ over 600 °C. Consequently, the optimum gasification temperature in 65%CO_2_/35%O_2_ and 50%CO_2_/50%O_2_ atmospheres was around 500 °C, which was lower than that in low-O_2_ environments. Summarily, the optimum temperature showed an overall relationship with the reactive agent, i.e., the optimum temperature decreased with increasing oxygen concentration, which was of great significance for a reasonable selective of gasification condition. It should be pointed out that there could be some water remaining in the sludge before gasification utilization in a real system, which would consume heat because of its vaporization; thus the temperature should be properly adjusted in the practical industrial process.

In addition, the effect of sludge/slags ratios could also be figured out from Fig. 4. As can be observed, the influence of the sludge/slags ratios on the syngas production could be ignored in atmospheres of 80%N_2_/20%O_2_, 80%CO_2_/20%O_2_ and 65%CO_2_/35%O_2_; while in an atmosphere of 50%CO_2_/50%O_2_, the syngas production remarkably increased with the addition of slags. The increasing oxygen concentration enhanced the char combustion, which could cause more heat production and CO_2_ formation and therefore less CO and H_2_ release. Meanwhile, the addition of slags could restrict the contact between the reactive agent and the sludge, especially the contact between the reactive agent and the formed char. Therefore the heterogeneous reaction between char and the oxygen was remarkably suppressed, and consequently, less CO_2_ could form and more CO and H_2_ were produced.

Moreover, it can be noted that in CO_2_/O_2_ atmospheres, the oxygen concentration greatly influenced the syngas yield at 400–800 °C; i.e., with increasing oxygen concentration, the amount of syngas production was substantially decreased, overall. This phenomenon could generate from two factors. Firstly, as aforementioned, the increasing oxygen concentration enhanced the char combustion, resulting in more CO_2_ formation and therefore less CO and H_2_ release. Actually, the phenomena that oxygen enhanced volatile release and benefitted ignition has also been observed in other studies^21^,^25^. Secondly, the reaction between CO_2_ gas and sludge could also produce more CO. As aforementioned, the trend of increasing syngas production with increasing CO_2_ concentration provided a clue of CCS, which was one of the most important objectives of the sludge/CO_2_ gasification.

### Leaching of toxic elements in the mixture of sludge ash and slags

To study the potential utilization of mixtures of the solid residuals after gasification, the leaching behaviors of the toxic elements (As, Zn, Cr, Mn and Ga) from pure slags, pure sludge ash for sample **SS1**, and the mixed solid residuals for sample **SS3** (Gasified at 800 °C) were explored and the concentrations of these elements in the leachate are detected (Supplementary Fig. S1a & S1b). It was found that the overall concentrations of these elements in the mixture were lower than those calculated according to the linear additive role of slag and ash, indicating that there was some synergy effect between slag and ash for stabilization and immobilization of the toxic elements. It has been also reported that sintering exhibited good binding capacity for the HMs in ceramsite^32^,^33^, and a mixture of the sludge ash and slags enhanced the capacity. The increasing stability of these toxic elements would relieve the negative impact of the solid residuals on the environment and therefore make up a great advantage of the further application of the solid residuals as cementitious materials. The morphology of the mixtures by scanning electron microscope (SEM) showed a state of homogeneous mixing and the measures results by the energy dispersive X-ray spectroscopy (EDS) showed that not only the Ca and Al elements but also the S and Cl elements from sludge were uniformly distributed in the solid residuals (Supplementary Fig. S1c & S1d), which indicated that the slags and sludge ash fully cohered in the solid residuals, which lead to the synergetic effect of the immobilization of the toxic elements.

Besides, during the gasification process, another interesting phenomenon was observed; the release of SO_2_ substantially decreased with the addition of slags. For example, when the gasification was conducted at 800 ^°^C in 80%CO_2_/20%O_2_ atmosphere, the maximum concentration of SO_2_ for sample **SS1** was 195 ppm, while that for sample **SS3** reduced to 120 ppm (Supplementary Fig. S2), suggesting that the CaO in the slags captured SO_2_^34^ and therefore the slags showed some effect of sulfur retention, which was also in consistent with the homogeneous composition of the solid residuals.

## Discussion

As aforementioned, the process of syngas release could be divided into three stages; i.e., volatile release, char transformation and fixed carbon reaction. The syngas release during different stages could be expressed by the corresponding Gaussian distributions, based on which the kinetics of these three stages could be obtained. The common kinetic equations of differential and integral types can be generally described as follows^27^,^35^,^36^:


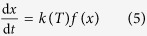



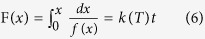


where 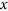 is the conversion degree, 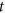 is the time, 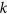 is the rate constant, 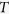 is the absolute temperature, 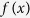 and 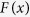 are the differential and integral mechanism functions (The kinetic functions are shown in Supplementary Table S1).

**Figure 5:**
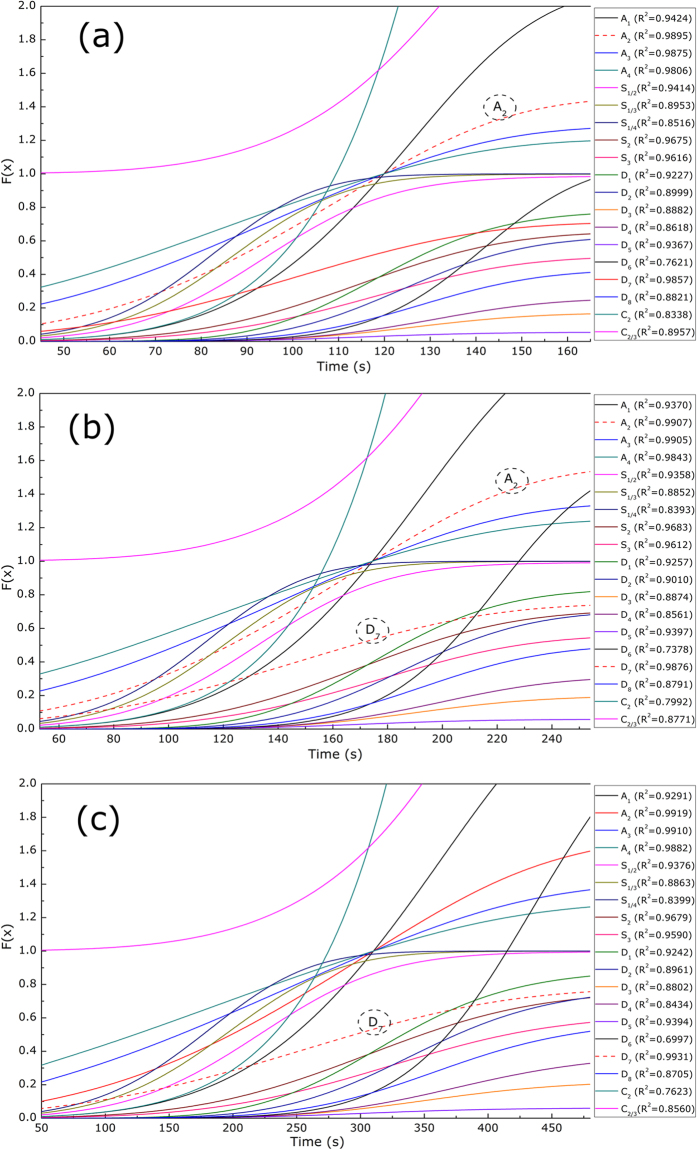
Model fittings of versus time during gasification at 500^o^C in 80%CO_2_/20%O_2_ atmosphere for samples **SS1** (**a**) Stage 1, (**b**) Stage 2 and (**c**) Stage 3.

By plotting 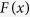 versus 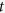 and analyzing the linear relationship between them using various mechanism functions, the most probable mechanism model could be obtained. As an example, Fig. 5 shows the experimental plots of 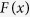 versus time during gasification at 500 °C in 80%CO_2_/20%O_2_ atmosphere for samples **SS1**. It was found that the experimental data at the stage of volatile release could be best reproduced by an A_2_ model (Avrami–Erofeev, m = 2) (R^2^ = 0.9895), which is applicable to materials whose porosity changes during the reaction, such as the gasification of biomass with high content of volatile^27^,^29^. The experimental data at the stage of fixed carbon reaction could be best interpreted by a D_7_ model (3-D, Jander) (R^2^ = 0.9931). While for the stage of char transformation, both the A_2_ model and the D_7_ model well fitted the experimental data, which might suggest that the mechanism function of char transformation was a mixture of A_2_ and D_7_ model.

**Figure 6:**
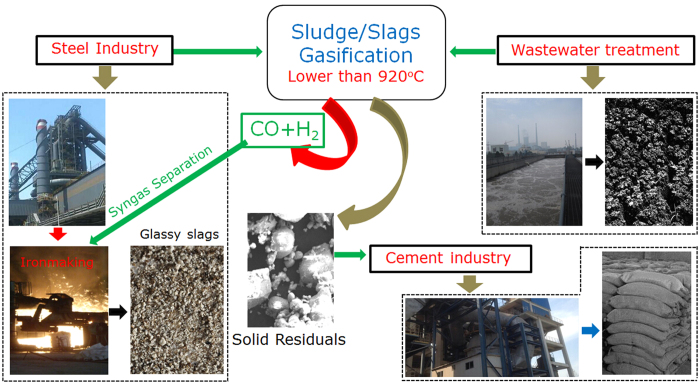
An integrated concept of the proposed prototype. All photographs and images were produced by Y. Q. Sun.

The syngas (CO + H_2_) produced by sludge gasification could be utilized for DRI (direct reduced iron) production^37^, which may provide a pathway to improve the energy efficiency and reduce the CO_2_ emission in the steel industry. Based on the sludge/slag gasification, an industrial prototype made up of multiple industrial and urban systems could be proposed, as depicted in Fig. 6. The whole prototype was composed of several processes. Liquid slags are first produced in the steel industry, granulated into small droplets with the diameter of <3 mm^3^,^4^, and then rapidly quenched to the temperature lower than 920 °C to form small glassy slags. Then the hot glassy slags are contacted with the ground dry sludge to perform the gasification reaction. Temperatures of the bulk mixture are determined by the sludge/slag ratio and individual temperatures. The produced syngas is separated, purified, and subsequently applied for DRI production, and meanwhile, the produced glassy slags and sludge ash can be used for cement manufacture. Finally, both the waste heat in slags and the material resources of sludge and slags are recovered.

In addition, the potential of energy savings and emission reduction of this prototype based on sludge/slag gasification can be calculated, as listed in Table 1. The reference of the calculation was that the high temperature slags were naturally discharged with a waste of sensible heat and meanwhile the sludge was combusted without resource recycling. According to Fig. 4, the gasification of sample **SS2** (sludge/slag = 1:1) at 600 °C in 80%CO_2_/20%O_2_ atmosphere produced the most syngas, i.e., 87.03 Nm^3^ CO, and 37.41 Nm^3^ H_2_ per ton of dry SS. If a large steel plant in China produces 10 million t steel per year, 3 million t slags would be generated and, if sludge/slag ratio is assumed to be 1:1, 261.09 million Nm^3^ CO and 112.23 million Nm^3^ H_2_ would be produced through sludge gasification, which can transform 8.14*10^5^ tons of Fe_2_O_3_ into 5.70*10^5^ tons of Fe. Certainly, the produced syngas should be reasonably separated and purified for the further utilization; the obtained CO was subsequently used for DRI production and the separated H_2_ could be utilized for hydrogen metallurgy. Therefore the potential annual energy savings at this steel plant would be as large as 1.83*10^5^ tons of standard coal and the potential reduction of CO_2_ emissions would be 6.72*10^5^ tons per year. It should be pointed out that there are three more factors that would further reduce the CO_2_ emission. First, some CO_2_ was consumed during sludge gasification process in CO_2_/O_2_ atmosphere, which was a prominent part of CCS. Second, the glassy slags and sludge ash could be used in the cement industry to substitute CaO (generally produced by CO_2_-generating CaCO_3_ calclination^4^,^7^), which could also contribute to an energy saving of 1.48*10^5^ tons of standard coal and an emission reduction of 1.07*10^6^ tons of CO_2_. Third, with residual steam the production of CO and H_2_ was increased. For example, when gasifying with 10% steam at 600 °C, the production of CO and H_2_ increased from 0.0838 L and 0.0377 L to 0.0844 L and 0.0392 L, respectively, per gram of sludge (**SS1**) (CO_2_:O_2_ = 4:1). Additionally, the syngas production for sample **SS1** gasified at various temperatures was listed in Supplementary Table S2 in atmosphere of 72%CO_2_/18%O_2_/10%steam. Furthermore, if, to produce dried sludge feedstock, SS could be treated using slag heat, more energy and CO_2_ emission would be saved. According to the foregoing results, there is a great potential of energy efficiency improvement and CO_2_ emission reduction in the steel industry using sludge/slag gasification. Most importantly, the introduction of this integrated system not only requires the individual technologies but also requires the collaboration of different sectors. In other words, a reasonable city plan is the precondition for urban acceptance of this integrated system or even an eco-city in the near future^38^,^39^.

In summary, we explored an emerging method of waste heat recovery from hot slags for syngas production, namely isothermal sludge gasification at 400–800 °C in N_2_/O_2_ and CO_2_/O_2_ atmospheres. It was determined that the hot slags can act as heat carrier for sludge gasification at 400–800 °C. Sludge gasification process could be divided three stages, namely volatile release, char transformation and fixed carbon reaction by Gaussian fittings. Compared with the N_2_/O_2_ atmosphere, the syngas production (CO + H_2_) in the CO_2_/O_2_ atmosphere was larger, and the increasing CO_2_ concentration improved the formation of CO and H_2_, which provided a potential method of CCS. The produced syngas can be used for DRI production or hydrogen metallurgy, which could substantially reduce the energy consumption and CO_2_ emission in the steel industry. Accordingly, an industrial prototype with multiple systems was put forward, in which both the waste heat in slags and the material resources of slags and sludge were recovered.

## Methods

### Sample preparation

The sludge sample was collected from a municipal wastewater treatment plant in Beijing, China. This sludge from municipal sewage contained a certain amount of toxic elements including sulfur and heavy metals, which should be fixed during the subsequent disposal process to reduce the environmental pollution. The results of ultimate and proximate analyses and the composition of sludge ash are listed in Table 2, according to which the chemical formula of the raw material is calculated as CH_1.82_O_0.77_. The sludge was first dried in air at 105 °C for 24 h, and then crushed into particle size less than 200 meshes using a rotary cutting mill. Water quenched BFS, produced from Shougang Corporation, China, were used and the chemical compositions were analyzed by X-Ray fluoroscopy (XRF, S4-Explorer, Bruker), as listed in Table 2. BFS alone account for more than 70% energy of slags^4^ and therefore this study concentrated on BFS. The slags were first dried in air at 105^o^C for 24 h, and then crushed and ground to 200 meshes, the glassy state of which was confirmed by X-ray diffraction (XRD, D/Max 2500, Rigaku) technique (Supplementary Fig.S3). It should be pointed out that water quenched slags were used in this study to perform the experiments in order to simulate the practical process and confirm the further utilization as cementitious materials. Then these ground slags and sludge were thoroughly mixed to perform the gasification experiments.

### Cooling path analysis of BFS

In order to identify the cooling path of the slags, isothermal experiments were performed using SHTT for visualizing phase transformation and measuring crystallization behaviors of slags. The working mechanism and schematic diagram of SHTT has been described elsewhere in detail^40^ and was briefly introduced here. The slag was first heated to 1550 °C for melting and held for 120 s to homogenize the chemical compositions and eliminate the bubbles. The liquid slag was then rapidly quenched to a holding temperature, and it took some time to crystallize, defined as incubation time. Incubation time, in part, represents the crystallization ability of slags, which lay the scientific foundation for heat recovery and the further utilization of slags.

### Gasification setup

In this study, isothermal gasification experiments were performed using a fixed bed system mainly involving a reactant gas control part, a tube furnace reactor, a gas condenser and a purifier and a gas analyzer (Supplementary Fig.S4). A quartz boat was used to hold the reactants. The reactive atmosphere was a synthetic gas mixture of CO_2_/O_2_ or N_2_/O_2_, which has been widely used to incinerate the sludge^21^,^24^,^25^. The gasification temperatures were chosen as 400, 500, 600, 700 and 800 °C, taking fully account of both the cooling path of slags and the sludge thermochemical process. The flow rate of reactive atmospheres was 200 ml/min with the gas content of 80%CO_2_/20%O_2_, 65%CO_2_/35%O_2_, 50%CO_2_/50%O_2_ and 80%N_2_/20%O_2_. The present gasification reactions were actually performed under a non-equilibrium condition where the produced CO and H_2_ were quickly taken away by the continuous gasifying agent and the consumed reactive agent was greatly less than the required one for stoichiometric combustion. During gasification process, the quartz boat filled with samples was loaded on the left side of the tube and then the reactive atmosphere was pumped into the system to fully expel the air inside. When the temperature reached the set point and was maintained for a period of time, the quartz boat was rapidly pushed into the middle of the tube to initiate the gasification reaction. After purification by the gas condenser and purifier, the volume and composition of the syngas was detected by the gas analyzer (Testo pro350, Testo).

Furthermore, to confirm the utilization of the solid residuals after gasification, the leaching behavior of the toxic elements was identified using Toxicity Characteristic Leaching Procedure (TCLP)^41^ in addition with water-leaching tests with a solid to liquid (S/L) ratio of 1/20 g/ml and a treatment time of 18 h; the concentration of the toxic elements in the solution were detected by Ion Coupled Plasma Mass Spectrometer (ICP-MS, 1-800-LEEMANS, Teledyne Leeman Labs). Meanwhile the morphology of the solid residuals was obtained by scanning electron microscope SEM with EDS (Hitachi S4800, Bruker).

## Additional Information

**How to cite this article**: Sun, Y. *et al*. Achieving waste to energy through sewage sludge gasification using hot slags: syngas production. *Sci. Rep*. **5**, 11436; doi: 10.1038/srep11436 (2015).

## Supplementary Material

Supplementary Information

## Figures and Tables

**Table 1:** Potential energy saving and emission reduction by the proposed prototype.

Resource	Reaction	Energy saving (t standard coal)	CO_2_ emission reduction (t)
Syngas	3CO + Fe_2_O_3_ = 2Fe + 3CO_2_ 3H_2_ + Fe_2_O_3_ = 2Fe + 3H_2_O	1.83*10^5^	6.72*10^5^
Glassy slags	CaCO_3_ = CaO + CO_2_	1.24*10^5^	8.93*10^5^
Sludge ash	CaCO_3_ = CaO + CO_2_	2.44*10^4^	1.76*10^5^
Sum		3.31*10^5^	1.74*10^6^

**Table 2:** Characteristics of SS and BFS.

	Proximate analysis/%				Ultimate analysis/%					HHV(j/g)
	Moisture	Volatile	Ash	Fixed carbon	C	H	O	N	S	
SS	1.97	36.53	54.34	9.13	22.11	3.37	22.92	4.97	1.07	9345.92
	CaO	SiO_2_	MgO	Al_2_O_3_	Fe_2_O_3_	Na_2_O	TiO_2_	MnO	K_2_O	
BFS (XRF)	37.88	34.61	15.62	8.56	0.60	0.54	0.45	0.36	0.26	
	SiO_2_	Al_2_O_3_	CaO	P_2_O_5_	Fe_2_O_3_	MgO	Na_2_O	K_2_O	TiO_2_	
Sludge ash (XRF)	34.44	18.14	13.77	13.02	8.01	5.69	2.43	2.34	0.661	
