# Supplementary material for: Achieving waste to energy through sewage sludge gasification using hot slags: syngas production
Source: Sci Rep. 2015 Jun 15;5:11436. doi: 10.1038/srep11436 (PMC4466782; doi:10.1038/srep11436)

Achieving waste to energy through sewage sludge gasification using hot slags: syngas production

Supplementary Information

Yongqi Sun[1], Jinichiro Nakano[2], Lili Liu[1], Xidong Wang[1,3] and Zuotai Zhang[1,3*]

[1]Department of Energy and Resources Engineering, College of Engineering, Peking University, Beijing 100871, P.R. China. [2]URS Corp., PO BOX 1959, Albany, OR 97321, USA. [3]Beijing Key Laboratory for Solid Waste Utilization and Management, College of Engineering, Peking University, Beijing 100871, P.R. China

Supplementary Table S1. Common Gas-Solid reaction mechanism functions. For a specific kind of kinetic model, m represents the integral exponent and n represents the reaction order.

No	Reaction mechanism	Differential function: f(x)	Integral function: F(x)
A_m	Avrami-Erofeev	$m(1-x)[-\ln(1-x)]^{m-1/m}$	$[-\ln(1-x)]^{1/m}$
A_1	m=1	$1-x$	$-\ln(1-x)$
A_2	m=2	$2(1-x)[-\ln(1-x)]^{1/2}$	$[-\ln(1-x)]^{1/2}$
A_3	m=3	$3(1-x)[-\ln(1-x)]^{2/3}$	$[-\ln(1-x)]^{1/3}$
A_4	m=4	$4(1-x)[-\ln(1-x)]^{3/4}$	$[-\ln(1-x)]^{1/3}$
S_m	Shrinking core	$m(1-x)^{m-1/m}$	$1-(1-x)^{1/m}$
$S_{1/2}$	m=1/2	$(1/2)(1-x)^{-1}$	$1-(1-x)^{2}$
$S_{1/3}$	m=1/3	$(1/3)(1-x)^{-2}$	$1-(1-x)^{3}$
$S_{1/4}$	m=1/4	$(1/4)(1-x)^{-3}$	$1-(1-x)^{4}$
S_2	m=2	$2(1-x)^{1/2}$	$1-(1-x)^{1/2}$
S_3	m=3	$3(1-x)^{2/3}$	$1-(1-x)^{1/3}$
D_m	Diffusion model		
D_1	one-dimensional	$1/2x^{-1}$	x^2
D_2	two-dimensional	$[-\ln(1-x)]^{-1}$	$x+(1-x) \ln(1-x)$
D_3	three-dimensional	$(3/2)(1-x)^{2/3}[1-(1-x)^{1/3}]^{-1}$	$[1-(1-x)^{1/3}]^2$
D_4	three-dimensional	$(3/2)[(1-x)^{-1/3}-1]^{-1}$	$1-2/3x-(1-x)^{2/3}$
D_5	3-D (anti-Jander)	$(3/2)(1+x)^{2/3}[(1+x)^{1/3}-1]^{-1}$	$[(1+x)^{1/3}-1]^2$
D_6	3-D (ZLT)	$(3/2)(1-x)^{4/3}[(1-x)^{-1/3}-1]^{-1}$	$[(1-x)^{-1/3}-1]^2$
D_7	3-D (Jander)	$6(1-x)^{2/3}[1-(1-x)^{1/3}]^{1/2}$	$[1-(1-x)^{1/3}]^{1/2}$
D_8	2-D (Jander)	$(1-x)^{1/2}[1-(1-x)^{1/2}]^2$	$[1-(1-x)^{1/2}]^2$
C_n	Chemical reaction	$(1-x)^n$	$(1-(1-x)^{1-n})/(1-n)$
C_2	n=2	$(1-x)^2$	$(1-x)^{-1}-1$
$C_{3/2}$	n=3/2	$2(1-x)^{(3/2)}$	$(1-x)^{-1/2}-1$

Supplementary Table S2. Syngas production in atmosphere of 80%CO_2/20%O_2 and 80%CO_2/20%O_2 with 10% steam (sample **SS1**).

Atmosphere	Syngas production (L/g_{sludge})	400°C	500°C	600°C	700°C	800°C
80%CO_2/20%O_2	CO	0.0470	0.0789	0.0838	0.0561	0.0395
	H_2	0.0165	0.0264	0.0377	0.0312	0.0275
72%CO_2/18%O_2/10%Steam	CO	0.0460	0.0813	0.0844	0.0584	0.0473
	H_2	0.0172	0.0298	0.0392	0.0322	0.0310

Supplementary Figure S1. Leaching behavior and morphology of the mixtures of solid residuals after gasification: (a) TCLP, (b) Water leaching, (c) SEM, and (d) EDS (Gasified at 800 ^{o}C)

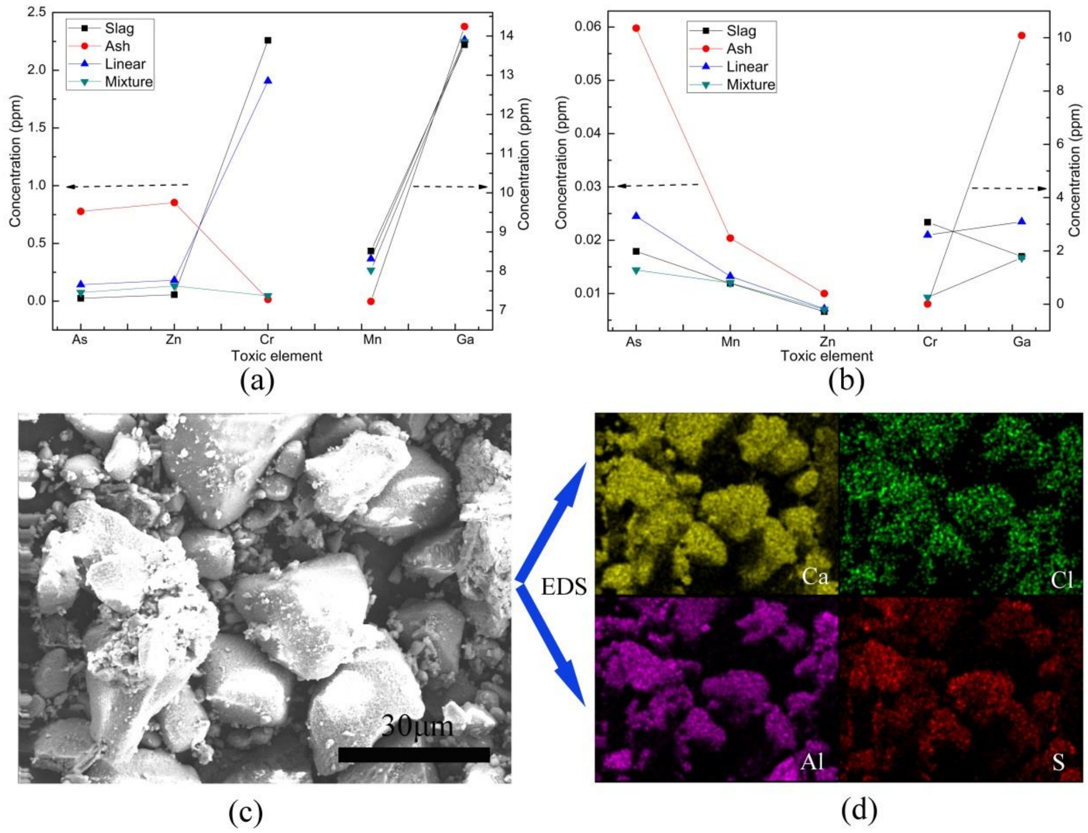

Supplementary Figure S2. SO_2 release during the sludge gasification at 800 ^oC in 80%CO_2/20%O_2 atmosphere.

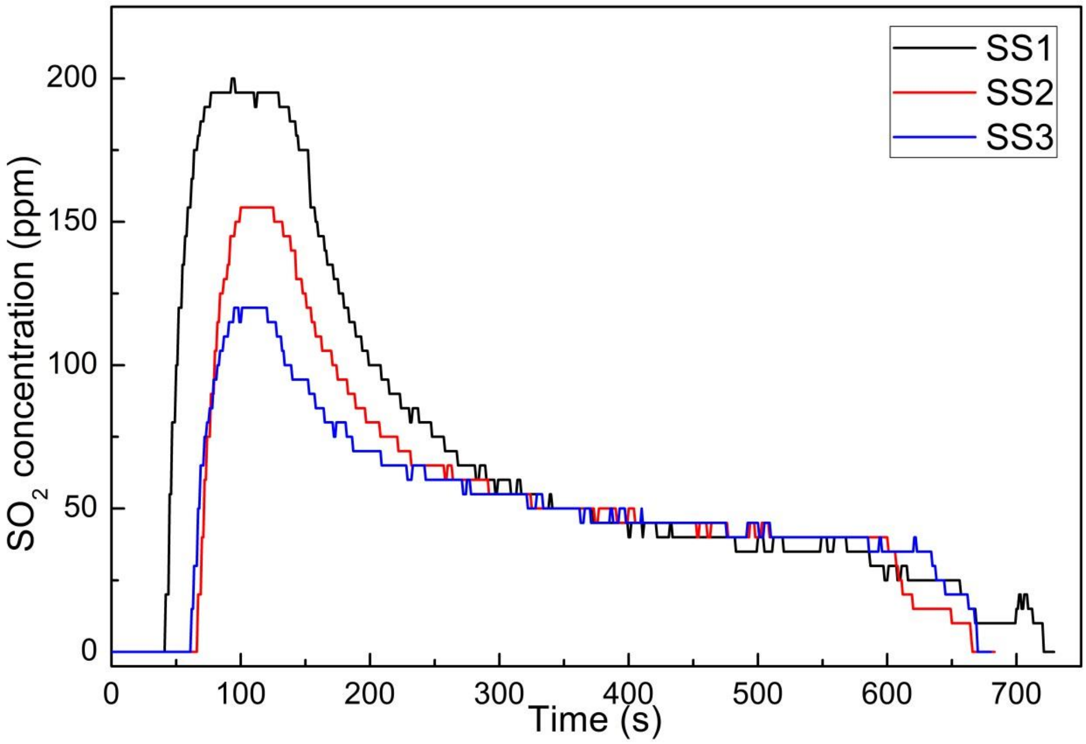

Supplementary Figure S3. XRD results of the used water-quenched industrial BFS from Shougang Corporation, China.

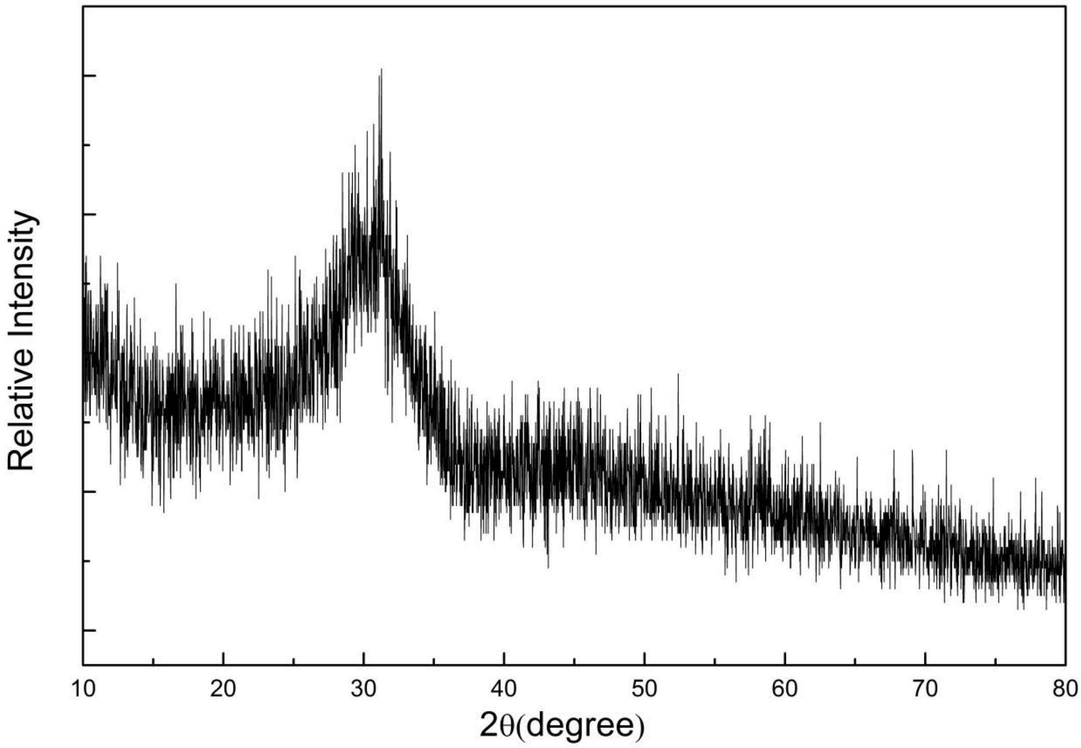

Supplementary Figure S4. Schematic of sludge gasification system: a quartz tube reactor, externally heated by an electric furnace, was used with the length of 110 mm and the inner diameter of 30 mm.

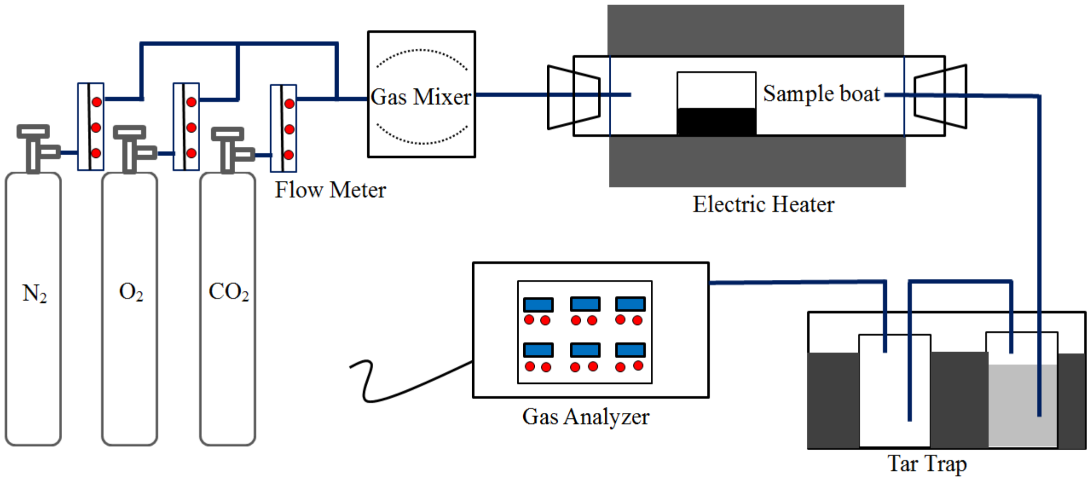

Supplement: Supplementary Information [file srep11436-s1.pdf]
